# A Multicentre Randomized Clinical Trial on Efficacy and Safety of Huxin Formula in Patients Undergoing Percutaneous Coronary Intervention

**DOI:** 10.1155/2014/143064

**Published:** 2014-05-26

**Authors:** Huan-Lin Wu, Yun-Fei Wang, Jun-Zhe Li, Min-Zhou Zhang, Xiao-Gang Sheng, Xia Wang, Song Li, Qiu-Xiong Chen, Xiao-Qing Li, Ai-hua Ou, Xin-Min Ruan

**Affiliations:** ^1^Heart Center, The Second Affiliated Hospital of Guangzhou University of Chinese Medicine, Guangzhou 510020, China; ^2^Department of DME, Guangzhou University of Chinese Medicine, Guangzhou 510020, China

## Abstract

Percutaneous coronary intervention (PCI) is widely used in clinical treatment of coronary artery disease. However, the effects of PCI on preventing restenosis after revascularization and improving the quality of life were not satisfying. Huxin Formula is formulated by modifying an experienced Chinese medicine formula and has been widely used in clinical practice due to its marked effects on coronary heart disease. A multicentre double-blind randomized controlled clinical trial was designed to evaluate the effects and safety of Huxin Formula in patients undergoing PCI. Our results showed that there was no significant difference between the two groups in main outcomes. For patients with ejection fraction (EF) >50%, score of the quality of life scale was higher in treatment group compared with control group. For patients with unstable angina, score of the quality of life scale in 360 days was significantly higher in treatment group compared with control group (*P* < 0.05). No obvious adverse reaction was found in the use of Huxin Formula. In conclusion, Huxin Formula, believed to be a safe treatment for patients after PCI, has benefits in improving the quality of life in patients with unstable angina though it failed to show superiority in primary and secondary outcomes.

## 1. Introduction

Coronary heart disease causes huge damage in human health and lives. Percutaneous coronary intervention (PCI) and coronary artery bypass grafting (CABG), which greatly reduce the incidence of cardiovascular events and alleviate the symptoms, are widely used in clinical treatment since the development of modern medicine. However, the effects of both treatments on preventing restenosis after revascularization and improving the quality of life were not satisfying. Recent studies had showed that traditional Chinese medicine (TCM) has advantages in preventing restenosis after PCI and improving the quality of life, but these trials had limited internal validity due to poor design and small sample size.

Huxin Formula is formulated by modifying an experienced Chinese medicine formula which is created by a well-known Chinese doctor, Professor DENG Tie-tao, and has been widely used in clinical practice due to its marked effects on coronary heart disease.

In this trial, we evaluated the efficacy and safety of Huxin Formula in patients after PCI using a multicenter, double-blind, randomized, and placebo-controlled clinical trial.

## 2. Methods

### 2.1. Subjects

Participants were screened by investigators at 12 first-rate domestic hospitals in China, including Guangdong Provincial Hospital of Chinese Medicine, Guangdong Provincial People's Hospital, Xinjiang Hospital of Chinese Medicine, Jiangsu Provincial Hospital of Chinese Medicine, The No. 10 Hospital of Shanghai, Shanxi Provincial People's Hospital, Gansu Provincial People's Hospital, The First Affiliated Hospital of Sun Yat-sen University, The First Affiliated Hospital of Guangzhou University of TCM, Huaqiao Hospital of Jinan University, Jiangmen Wuyi Hospital of TCM, and People's Hospital of Liuzhou. The study was conducted between April 2008 and December 2010. Patients were assessed according to ESC 2006 guidelines for management of patients with stable angina and ACC/AHA 2005 guidelines for management of patients with unstable angina/non-ST-elevation myocardial infarction. The inclusion and exclusion criteria are shown in Table 1. Patients were free to withdraw from the study at any time. Potential participants who were interested in this study received a complete explanation of the protocol and signed the consent form. The ethical approval for the study was granted by the Ethics Committee of Guangzhou University of Traditional Chinese Medicine.

### 2.2. Design, Randomization, and Allocation

A double-blind randomized controlled clinical trial was conducted by using stratified randomized method. Center is a stratified factor in the present trial. Eligible participants after PCI were randomly assigned into 2 groups: treatment group who received conventional treatment and Huxin Formula or control group who received conventional treatment and the placebo. A sample size calculation based on the incidence rate of cardiovascular events in previous studies determined that 289 patients were needed to reduce the incidence rate of cardiovascular events of 8.5% (power 0.85, significance level 0.05). With an estimated 15% dropout rate, we set the total sample size at 680. Randomized codes were performed with SAS6.12 (statistical software package UNIFORM(n)). Eligible patients at each center were assigned randomization numbers from Institute of Basic Medical and Clinical Sciences, China Academy of Chinese Medical Sciences, which is responsible for the randomization of this research and was external to the trial. Participants and investigators were masked to group assignment. The prepared drugs were dispensed in similar looking bags, and the nature of the drug was concealed by the providers. The random allocation sequence was concealed until the data collection for the entire study was completed.

### 2.3. Treatment Protocols

Patients in the treatment group were provided Huxin Formula (in the form of granules) prepared by Jiangyin Pharmaceutical Co., Ltd. (Jiangsu, China). Every 10 g Huxin Formula granules consisted of ginseng (10 g), Exocarpium Citri Rubrum (5 g),* Panax pseudoginseng* (8 g),* Pinellia ternata* (10 g),* Salvia miltiorrhiza* (10 g), and* Agastache* (10 g) as crude drug. Patients in the control group were given placebo granules prepared by the same supplier. Placebo granules were designed to taste, smell, and look similar to Huxin Formula. Placebo consisted of amylum, bitter principle, excipient, and so forth. Huxin Formula or placebo was used once a day (10 g QD) after fully dissolving in 300 mL of boiled water cooled to 70°C. Treatment started in one week after PCI and continued for 6 months (180 days). Then patients were followed up for another 6 months (180 days).

### 2.4. Outcomes

The baseline data, including gender, age, personal history, medical history, family history of cardiovascular disease, classification of cardiac function, and clinical classification of coronary heart disease, were assessed. Main cardiovascular events (death, nonfatal myocardial infarction, and repeat revascularization) were also observed and recorded. We assessed quality of life using two scales: (1) Seattle Angina Questionnaire (SAQ) and (2) scale of the life quality in integrative medicine for CAD (see Supplementary Material available online at http://dx.doi.org/10.1155/2014/143064). The outcomes were assessed at 0, 90, 180, 270, and 360 days after treatment.

### 2.5. Safety Monitoring

To assess the safety of the 6-month treatment, routine blood and liver and renal function tests were conducted before randomization, 90 days and 180 days after treatment. During the trial, adverse events were observed in detail and documented using case report forms.

### 2.6. Statistical Analysis

Statistical analysis sets included intention-to-treat (ITT) sets, per-protocol (PP) sets, and safety analysis sets. Descriptive analysis and inferential analysis of clinical features before and after treatment were as follows.

1. Measurement data: functional indexes are presented as mean ± standard deviation. Each group compared before and after treatment with paired *t*-test and Student's *t*-test was used to compare small sample data (including 95% confidence interval). Rank-sum test (Wilcoxon test) is used for nonnormal distribution or heterogeneity of variance. Analysis of covariance or stratified analysis is used when baseline is not neat.
2. Enumeration data: the constituent ratio and rate of each index were calculated. Two-group comparisons were performed using the fourfold table *χ* ^2^ test (or accurate probability method) for the total effective rate and the 2 × C table *χ* ^2^ test for the constituent ratio.
3. Ranked data: two-group comparisons were performed using Mann-Whitney test.
4. Life quality analysis: the general descriptive analysis is presented as mean ± standard deviation, maximum, minimum, and median.
5. The analysis of the factors affecting curative effect: the logistic regression analysis was used to analyze the factors that may affect the judgment of curative effect, including age, duration, classification of coronary heart disease, survival quality assessment scale of integrated traditional Chinese and western medicine, PCI operator, and center.
6. The Kaplan-Meier method was used to compare the cardiovascular event rates and median time. Influence factors of cardiovascular events were analyzed by using the Cox regression analysis.
7. Baseline comparison test level *α* = 0.10, and two-group effects comparison test level *α* = 0.05.

Safety analysis of each case was mainly performed as descriptive statistics, including incidence and description of adverse events, the changes of laboratory testing results before and after test, and the relationship between abnormal changes and investigational drugs.

## 3. Results

### 3.1. Participants Flow

**Figure 1:**
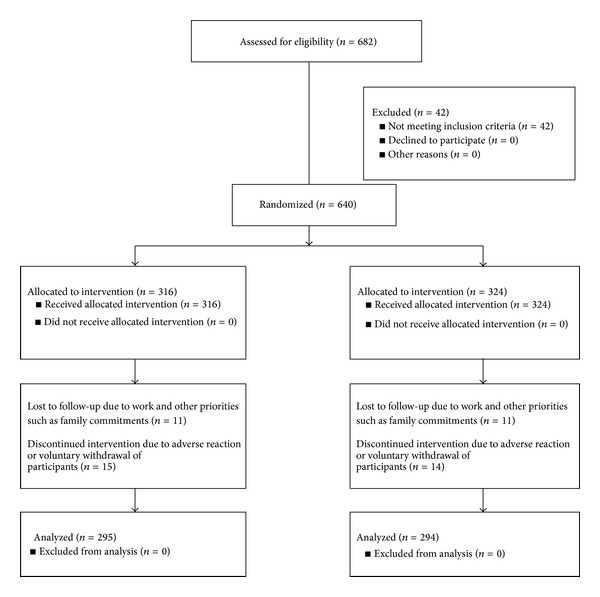
Consort 2010 flow diagram.

There were 682 patients with PCI treatment included in this research. 42 patients were eliminated, and 51 patients were withdrawn after 360 days. The flow of participants in the study is summarized in Figure 1.

### 3.2. Baseline Data

The general characteristics of the patients are shown in Table 2. The comparisons between control group and treatment group were performed using the chi-square test for baseline classification data before treatment, including the demographic data (gender, ethnic, and cultural), the general situation (height, pulse, respiration, heart rate, systolic blood pressure, and diastolic blood pressure), personal history (history of smoking, drinking, and allergy and family history of cardiovascular disease), medical history (dyslipidemia, high blood pressure, diabetes, stroke, heart failure, and arrhythmia), classification of cardiac function, and clinical classification of coronary heart disease (unstable angina and myocardial infarction). There were no significant deviations (*P* > 0.05) between the two groups except the weight (66.56 versus 64.82).

### 3.3. Major Endpoints

There were no significant differences (*P* > 0.05) between control group and treatment group in major end points at 360-day follow-up (Table 3), including mortality, number of patients with nonfatal myocardial infarction, proportion of further revascularization, and rehospitalization caused by cardiovascular events.

### 3.4. Secondary Outcomes

There were no significant differences (*P* > 0.05) between control group and treatment group on secondary outcomes at all time points after PCI, including angina scale (frequency of attack, duration time, intensity of pain, and dose of nitroglycerin), SAQ (dimension of body activities limitation, angina pectoris stable state, heart attacks, disease knowledge, and treatment satisfaction), and the quality of life scale of integrative medicine.

### 3.5. Stratified Analysis

#### 3.5.1. Stratified Analysis of EF before Treatment

As shown in Table 4, according to PCI per-protocol population and stratified analysis of EF before treatment, for patients with EF > 50%, score of the quality of life scale was higher but not statistically significant (*P* > 0.05) in treatment group compared with control group at all time points.

#### 3.5.2. Stratified Analysis of the Type of Coronary Heart Disease before Treatment

As shown in Table 5, according to PCI per-protocol population and stratified analysis of the type of coronary heart disease before treatment, for patients with unstable angina, score of the quality of life scale in 360 days was significantly higher in treatment group compared with control group (*P* < 0.05). There were no significant deviations (*P* > 0.05) between the two groups at another four time points.

### 3.6. Safety Analysis

Incidence of adverse event and specific event in treatment group was similar to control group. No obvious adverse reaction was found in the use of Huxin Formula.

## 4. Discussion

Coronary artery disease (CAD) is a major cause of mortality and morbidity in developed countries [1, 2]. Before developing the technique of PCI, coronary artery bypass graft (CABG) had been the standard and the only revascularization procedure. With the development of medical imaging and heart catheterization techniques, the PCI, as an effective, safe, less disabling, and less expensive revascularization procedure, had become a more frequently used treatment than CABG for CAD in most western countries as well as in China. Growing evidence had showed that PCI can improve myocardial ischemia and reduce the risks of long-term adverse cardiovascular events [3, 4]. The results of a meta-analysis of 13 randomized controlled trials showed that PCI with drug-eluting stents reduces the risks of major adverse cardiac events, recurrent myocardial infarction, and reintervention [5].

In recent years, other than biochemical endpoints, quality of life (QoL) is considered to be an important indicator of health outcome in CAD [6] and the most common indication for PCI [7]. Seattle Angina Questionnaire (SAQ) was the most commonly used instrument to capture comprehensive and sensitive changes in QoL of the cardiac patients [8–10]. Besides SAQ, scale of the life quality in integrative medicine for CAD, which was established for the first time, was used in the present research. The internal reliability of this scale was supported by the values of Cronbach's *α* that exceeded 0.7 for all the subscales. Scale of the life quality in integrative medicine for CAD was used to collect the mental and social functioning information that SAQ might have missed.

Although the role of PCI in chronic stable angina is well established as it alleviates ischemic symptoms and improves quality of life [11, 12], its benefit in general health status of patients with unstable angina and non-ST-elevation myocardial infarction (UA/NSTEMI) would not be sufficient. It is reported that poor QoL is highly prevalent in elderly patients undergoing PCI [13]. At 30 days after PCI, general health-related quality of life (HRQoL) was significantly lower (0.86 ± 0.21 versus 0.89 ± 0.17, *P* = 0.001) after adjusting baseline characteristics (*P* < 0.001) [14]. Therefore, in addition to angina-specific therapy, comprehensive supportive care would be needed to improve the quality of life, which might improve long-term clinical outcome especially in patients after PCI.

According to the 2007 ACCF/AHA guidelines for the management of patients with unstable angina/non-ST-elevation myocardial infarction [15], clopidogrel is recommend to be used for at least one year. Lipid-lowering therapy with the use of statin has benefits in blocking the progression of coronary artery plaque and further preventing cardiovascular events and death [16]. However, effects of these treatments on relieving the symptoms and improving quality of life are not satisfying.

It is reported that some kinds of herbal medicine, such as Xuefu Zhuyu Capsule [17], exhibit better efficacy on HRQoL in patients with unstable angina after PCI. However, the application of TCM in the post-PCI patients still lacks support from rigorously designed clinical trial. Huxin Formula is formulated by modifying an experienced Chinese medicine formula which is created by a well-known Chinese doctor, Professor DENG Tie-tao, and has been widely used in clinical practice because of its marked effects on coronary heart disease [18, 19]. Our earlier clinical study in 55 patients after CABG had showed that modified Huxin Formula can promote rehabilitation of the CABG patients, improving clinical symptoms and quality of life [20]. However, the effects of the formula in patients undergoing PCI were unknown. Therefore, the present study was designed to evaluate the efficacy and safety of Huxin Formula in patients after PCI.

Our results showed that, for patients with unstable angina, score of the quality of life scale in 360 days was significantly higher in treatment group (87.62 ± 7.52) compared with control group (85.70 ± 8.08) ( *P* < 0.05). This result suggested that Huxin Formula may improve the quality of life of patients after PCI compared to the placebo. There are 22 patients with the event of MACE (2 deaths, 6 repeat revascularizations, and 14 readmissions caused by cardiovascular events) in this research. The rate of one-year MACE event is only 3.23%, slightly lower than 5–8% that has been suggested in foreign related reports. There was no statistical difference between the two groups in other outcomes. Limited sample size and the relatively short observational period may be the two reasons for the negative results. Therefore, further studies with larger sample sizes and longer observational period are warranted on the base of these precious experiences.

## 5. Conclusion

In this research, the results showed that Huxin Formula is a safe treatment for patients after PCI. Though Huxin Formula failed to show superiority in primary and secondary outcomes, it has benefits in improving the quality of life in patients with unstable angina.

## Supplementary Material

Scale of the life quality in integrative medicine for CAD, which was established for the first time, was used in the present research. The internal reliability of this scale was supported by the values of Cronbach's *α* exceeded 0.7 for all the subscales. Scale of the life quality in integrative medicine for CAD was used to collect the mental and social functioning information that SAQ might have missed.

## Figures and Tables

**Table 1:** Inclusion and exclusion criteria.

Inclusion criteria (1) Age 40 to 75 without gender limitation (2) Patients who meet the diagnostic criteria of unstable angina or myocardial infarction (3) Patients who were diagnosed as “deficiency of heart-QI and phlegm stasis-bizu” according to traditional Chinese medicine (TCM) (4) One week after PCI Exclusion criteria (1) Patients who have acute myocardial infarction or severe heart failure (cardiac function class IV according to the cardiac function standard of New York Heart Association and the left ventricular ejection fraction ≤30% by heart color Doppler) (2) Patients who have severe liver and kidney dysfunction (serum alanine aminotransferase > three times the normal cap and/or plasma creatinine ≥442 umol/L) (3) Patients who have cancer or active gastrointestinal bleeding (4) Patients with mental illness (5) Patients who were pregnant or breast-feeding (6) Patients who have medical history of CABG and undergo PCI in vascular bridge (7) Patients with stable angina	

**Table 2:** Baseline clinical data (*n*, %).

	Control group (N = 316)	Treatment group (N = 324)	*P*
Age ($\bar{x}\pm s$)	59.67 ± 9.33	60.91 ± 8.97	0.09
Gender			
Male	249 (78.8)	250 (77.2)	0.62
Female	67 (21.2)	74 (22.8)	
Height ($\bar{x}\pm s$)	166.34 + 7.14	166.04 + 7.37	0.60
Weight ($\bar{x}\pm s$)	66.56 ± 8.89	64.82 ± 9.53	0.02
Pulse ($\bar{x}\pm s$)	74.6 ± 11.93	75.56 ± 11.92	0.31
Breath ($\bar{x}\pm s$)	19.52 ± 2.03	19.41 ± 1.69	0.46
Heart rate ($\bar{x}\pm s$)	74.71 ± 12.01	75.75 ± 12.29	0.28
SBP ($\bar{x}\pm s$)	129.17 ± 19.29	130.30 ± 22.00	0.49
DBP ($\bar{x}\pm s$)	77.21 ± 11.44	77.89 ± 12.58	0.47
Combined disease			
Dyslipidemia	96 (30.4)	91 (28.1)	0.52
Hypertension	182 (57.6)	179 (55.2)	0.55
Diabetes	72 (22.8)	60 (18.5)	0.18
Stroke	16 (5.1)	13 (4.0)	0.52
Heart failure	10 (3.2)	12 (3.7)	0.71
Gastrointestinal disorder	31 (9.8)	26 (8.0)	0.43
Arrhythmia	18 (5.7)	18 (5.6)	0.94
Medical history			
Smoking	159 (50.3)	165 (50.9)	0.88
Drinking	50 (15.8)	52 (16.0)	0.94
Family history of CVD	41 (13.0)	49 (15.1)	0.43
Allergy	31 (9.8)	30 (9.3)	0.81
Clinical classification of CHD			
Unstable angina	168 (53.2)	193 (59.6)	0.10
Ml	148 (46.8)	131 (40.4)	
Cardiac functions			
I	117 (37.0)	122 (37.7)	
II	157 (49.7)	166 (51.2)	0.70
III	42 (13.3)	36 (11.1)	

CHD: coronary heart disease, CVD: cardiovascular disease, DBP: diastolic blood pressure, MI: myocardial infarction, and SBP: systolic blood pressure.

**Table 3:** Major end points at 360 days.

Events		Control group (*n* = 295)	Treatment group (*n* = 294)	*χ* ^2^	*P* value
Death	Y	2 (0.7)	0 (0.0)	2.00	0.16
	N	293 (99.3)	294 (100.0)		

Nonfatal MI	Y	0 (0.0)	0 (0.0)	—	—
	N	295 (100.0)	294 (100.0)		

Repeat revascularization	Y	2 (0.7)	3 (1.0)	0.21	0.65
	N	293 (99.3)	291 (99.0)		

Readmission	Y	5 (1.7)	9 (3.1)	1.19	0.28
	N	290 (98.3)	285 (96.9)		

Values are *n* (%). MI: myocardial infarction.

**Table 4:** Score of the quality of life scale for patients with EF > 50% ($\overset{-}{x}\pm s$).

Time	Group	*n*	$\overset{-}{x}\pm s$	*Z*	*P*
0 day	Control group	244	71.80 ± 12.62	−0.24	0.81
	Treatment group	251	72.03 ± 12.18		

90 days	Control group	244	81.32 ± 9.592	−0.42	0.68
	Treatment group	251	81.79 ± 9.220		

180 days	Control group	244	82.84 ± 9.24	−0.67	0.51
	Treatment group	251	83.39 ± 8.58		

270 days	Control group	244	84.72 ± 8.77	−0.66	0.51
	Treatment group	251	85.26 ± 8.431		

360 days	Control group	244	86.29 ± 8.55	−1.04	0.30
	Treatment group	251	87.11 ± 7.70		

**Table 5:** Score of the quality of life scale for patients with unstable angina ($\overset{-}{x}\pm s$).

Time	Group	*n*	$\overset{-}{x}\pm s$	*Z*	*P*
0 day	Control group	168	69.61 ± 11.27	−0.32	0.75
	Treatment group	193	69.97 ± 10.65		

90 days	Control group	168	80.75 ± 8.94	−0.53	0.59
	Treatment group	193	81.09 ± 8.86		

180 days	Control group	168	82.44 ± 9.03	−1.06	0.29
	Treatment group	193	83.33 ± 8.11		

270 days	Control group	168	84.78 ± 8.18	−0.72	0.47
	Treatment group	193	85.23 ± 8.25		

360 days	Control group	168	85.70 ± 8.08	−2.51	0.01^#^
	Treatment group	193	87.62 ± 7.52		

^#^Significantly different versus control group (*P* < 0.05).

## Abbreviations

ACS: Acute coronary syndrome
BMS: Bare metal stents
CABG: Coronary artery bypass grafting
CAD: Coronary artery disease
CHD: Coronary heart disease
DES: Drug-eluting stents
EF: Ejection fraction
ITT: Intention-to-treat
MACE: Major adverse cardiovascular events
NSTEMI: Non-ST-elevation myocardial infarction
PCI: Percutaneous coronary intervention
PES: Paclitaxel-eluting stents
PP: Per-protocol
REVERSAL: Reversal of atherosclerosis with aggressive lipid lowering
SAQ: Seattle Angina Questionnaire
SES: Sirolimus-eluting stents
TCM: Traditional Chinese medicine
UA: Unstable angina.
