# Supplementary material for: A Multicentre Randomized Clinical Trial on Efficacy and Safety of Huxin Formula in Patients Undergoing Percutaneous Coronary Intervention
Source: Evid Based Complement Alternat Med. 2014 May 26;2014:143064. doi: 10.1155/2014/143064 (PMC4058264; doi:10.1155/2014/143064)
Supplement: Supplementary file 1 — Scale of the life quality in integrative medicine for CAD, which was established for the first time, was used in the present research. The internal reliability of this scale was supported by the values of Cronbach's α exceeded 0.7 for all the subscales. Scale of the life quality in integrative medicine for CAD was used to collect the mental and social functioning information that SAQ might have missed. [file 143064.f1.doc]

**Supplementary Material**

**Scale of the life quality in integrative medicine for CAD**


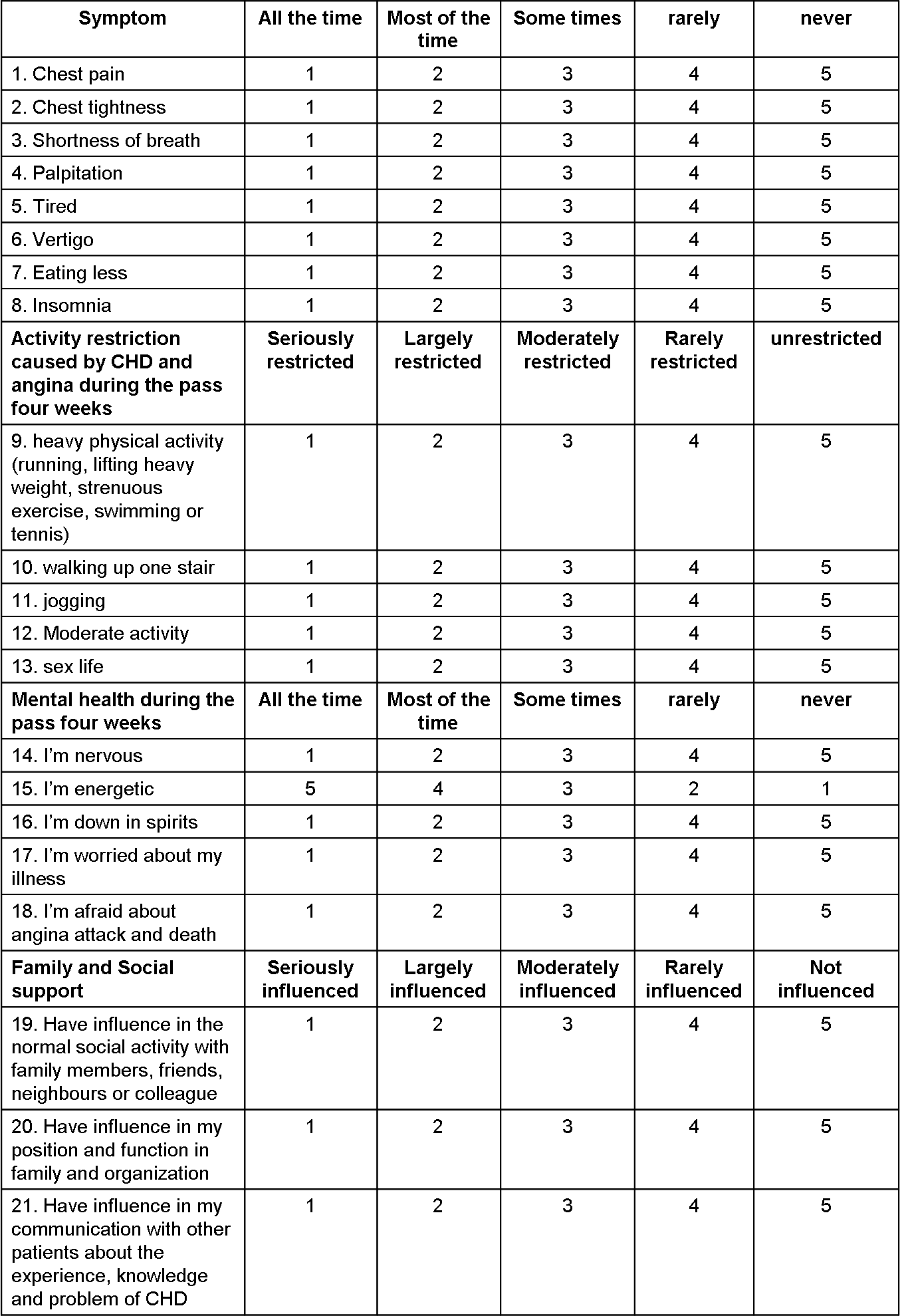


Final score=sums of each score / total score(105) ×100%
